# Micro‐Stimulation Timing Framed Around an Averaged Theta Period of Stimulation Determines Hippocampal Recruitment in Cued Fear Conditioning

**DOI:** 10.1002/hipo.70097

**Published:** 2026-04-19

**Authors:** Paula Gonçalves Vieira Teixeira, Leonardo de Oliveira Guarnieri, Grace Schenatto Pereira, Márcio Flávio Dutra Moraes

**Affiliations:** ^1^ Department of Physiology and Biophysics, Núcleo de Neurociências Federal University of Minas Gerais Belo Horizonte Brazil; ^2^ Center for Magnetic Resonance Technology and Research, Graduate Program in Electrical Engineering Federal University of Minas Gerais Belo Horizonte Brazil

**Keywords:** fear conditioning, memory‐trace, neuromodulation, phase‐coded microstimulation, phase‐resseting, temporal‐coding

## Abstract

The importance of precise timing of neuronal activity, relative to ongoing slower oscillations, is reshaping the engram theory and our understanding of how memories are encoded and stored. The hippocampal theta‐wave phase‐encoding of neuronal firing predicts behavioral outcomes and cognitive performance in memory tasks. A single external stimulus or a sensory/cognitive cue may induce Phase‐Resetting shift of theta waves, without changing their frequency or power. This phenomenon seems to be a core mechanism for temporal coordination, information encoding, and memory formation. We hypothesize that not only Phase‐Resetting, but temporally coded neuromodulation packaged around an averaged theta cycle of 140 ms, plays a role in engram formation. Inter‐pulse microstimulation patterns (MS) consisting of six stimuli within a 140 ms period were applied to the intermedial CA3 hippocampal area of C57/BL6 mice. Each MS‐pattern consisted of a 10‐bit word (each bit representing a 14‐ms bin), indicating the phase at which MS was applied. The randomized (MSr) or fixed pattern (MSf) stimulus was applied during a 30 s presentation of a pure tone (CS) that terminated with a 2 s/0.4 mA footshock (US). Sham animals underwent surgery and cued fear conditioning, but no MS. Cued fear memory was tested by presenting the CS (without MS) in a different context. The group of mice that received the MSf during conditioning showed higher levels of freezing compared to the Sham group; the MSr group did not. We measured c‐Fos/NeuN labeling as a proxy for neuronal activity 90 min after memory retrieval. As expected, since cued‐fear memory is predominantly amygdala‐dependent, all groups showed an increase in c‐Fos expression in the amygdala. However, only the MSf group had higher hippocampal activation after retrieval, suggesting that fixed pattern stimulation framed around an averaged theta cycle led to neuronal integration into the memory trace. Our findings indicate that temporal organization plays a crucial role in how memories are stored and accessed.

## 1 Introduction

Lesion studies have shaped the long‐standing view in neuroscience that classical “cued” fear conditioning is not significantly affected by damage to hippocampal structures but that it does impair context fear conditioning (Anagnostaras et al. 1999; Kim and Fanselow 1992; Maren et al. 1997). Additionally, amygdala lesions severely impair cued and context fear conditioning (LeDoux 1995; Phillips and LeDoux 1992). Although both forms of conditioning present an unconditioned stimulus (UCS—e.g., an intrinsic aversive stimulus) that is paired with a conditioned stimulus (CS—neutral before training) to provoke a conditioned response (CR) after training, the context is not a discrete CS. In contrast, the sound “tone” cue in the classic auditory fear conditioning paradigm serves as a particular and distinct CS, while context cues are dictated by the environment in which conditioning occurs during training (M. S. Fanselow 1981, 1984; Grillon, Baas, Cornwell, and Johnson 2006; Grillon, Baas, Pine, et al. 2006; Rescorla and Wagner 1972; Vansteenwegen et al. 2008). One initial corollary hypothesis that arises from the context conditioning impairment observed in hippocampus‐lesioned animals is whether hippocampal stimulation (i.e., microstimulation—MS neuromodulation), during cued fear conditioning paradigm (Otto and Poon 2006), with precautions to avoid context interference, could recruit hippocampal neuronal assemblies during memory evocation, therefore mimicking a cued/context conditioning protocol.

The contextual fear conditioning paradigm is one of the most well‐established models for studying hippocampal memory functions. A key aspect of forming memory representations within the hippocampus (i.e., memory traces) is the emergence of theta waves during memory acquisition and recall. Abolishing hippocampal theta, such as through medial septum lesions, impairs spatial memory (Winson 1978). Lipponen et al. (2012) attempted to restore hippocampal theta in fimbria‐fornix lesioned animals using electrical stimulation during the encoding phase (Lipponen et al. 2012). Although the artificially restored theta oscillation was similar to the endogenous theta rhythm in amplitude and frequency, it unexpectedly did not re‐establish contextual conditioned responses. It even impaired the contextual‐conditioned fear response in sham‐operated animals. This failure to elicit hippocampal recruitment with artificial theta stimulation may arise from a limited understanding, at the time, of the role of slow oscillations in temporal and phase encoding mechanisms (Ahmadi et al. 2025; Amil et al. 2024; Canavier 2015; Fell and Axmacher 2011; Ku et al. 2024), as well as their significance in long‐range integration among neuronal substrates. A brief paragraph on how neuroscience evolved in proposing a new brain architecture will help elucidate the apparent paradox and introduce the concept of temporal/phase coding in neural network representation.

When an external stimulus is applied to intrahippocampal structures it may realign the phase of ongoing hippocampal oscillations (most prominently theta), without necessarily changing their frequency or power (Canavier 2015; Givens 1996; Tass 1996). This phenomenon is termed Phase‐Resetting and it seems to be a core mechanism for temporal coordination, information encoding, and memory formation. This phenomenon has also been shown to occur with natural sensory input (sensory and/or cognitive tasks) causing the oscillation to jump to a new phase, synchronizing activity across neurons (Klimesch, Sauseng, Hanslmayr, Gruber, and Freunberger 2007). As mentioned before, the medium septum has a key role in theta formation and septal inputs can induce global theta phase resets, synchronizing large hippocampal populations. In addition, memory formation in the hippocampus seems to be tightly linked to the precise timing of neuronal activity relative to ongoing theta oscillations. One neuronal‐network plastic mechanism that requires very fine temporal constraints in order to promote its long‐lasting changes in network connectivity is the spike‐timing‐dependent‐plasticity (STDP) (Feldman 2012); which could provide a direct mechanistic bridge between phase‐dependent encoding and engram formation and retrieval. It has been shown that hippocampal pyramidal neurons exhibit enhanced dendritic depolarization, driven primarily by entorhinal cortex input, during encoding‐favorable phases of the theta cycle; thus, creating temporal windows in which presynaptic activity reliably precedes postsynaptic firing, a condition necessary for plastic changes under STDP rules (Bi and Poo 1998; Hasselmo et al. 2002). It has been shown that both LTP and LTD in the hippocampus may be modulated by theta phase, with potentiation preferentially occurring at specific phases that align spike timing within the STDP‐positive window (Huerta and Lisman 1995; Hyman et al. 2003). Attention‐based cognitive tests, in which salient or novel events are introduced, trigger Phase‐Resetting of hippocampal theta and enhances memory encoding. This enhancement seems to happen due to synchronizing neuronal populations and realigning spike timing to plasticity‐permissive phases, thereby increasing the likelihood that coincident activity is converted into stable synaptic changes (Klimesch, Sauseng, and Hanslmayr 2007; Klimesch, Sauseng, Hanslmayr, Gruber, and Freunberger 2007; Kota et al. 2020). Together, these findings support a unified framework in which theta phase organization and Phase‐Resetting shape hippocampal memory formation by constraining neuronal firing within the temporal rules of STDP, effectively translating oscillatory timing into long‐lasting synaptic modifications. However, it does not address the question if the information encoded within a theta cycle, and not only a single pulse Phase‐Reset stimuli, also plays a role in memory‐trace formation.

Our laboratory has employed temporally coded neuromodulation (i.e., micro‐stimulation—MS—in target areas) to create neuronal representations embedded in the underlying neuronal network—artificially generated engrams—while maintaining the same substrate being stimulated and the same overall stimulation frequency. Morão et al. demonstrated that varying interpulse patterns of MS targeting the amygdaloid complex, with specific phase encoding arrangements framed within an average theta wave period but keeping the same overall stimulation frequency, can generate distinct conditioned stimulus (CS) memory representations in a classical fear conditioning paradigm (Mourão et al. 2016). Furthermore, neuromodulation that differs solely in its temporal/phase encoding could recruit different neural pathways, highlighting the functional connectome of large‐scale integration within the brain. In this work, we aimed to evaluate whether interpulse patterns of MS framed within an average theta wave period—temporally coded stimulation—play a crucial role in recruiting hippocampal structures during a hippocampus‐independent classical cued fear conditioning task, as opposed to hippocampus‐dependent contextual fear paradigms. In order to evaluate the role of the temporally coded stimuli within a theta wave and, not only the Phase‐Resetting mechanism of theta synchronization, we started and ended all packaged‐stimulation words framed within an average theta period with a stimuli. In other words, each stimulus consisted of a 10‐bit coded word (each bit representing a 14‐ms bin within a theta wave; 140 ms ~7 Hz), indicating the phase at which MS was administered and, we applied either a randomized (MSr) or fixed (MSf) theta micro‐stimulation pattern to the hippocampus during the acquisition phase of a classic auditory fear conditioning task. We hypothesize that only neuromodulation using fixed temporally coded patterns could engage the hippocampus within the process of memory neural representation and, therefore, be evoked by the cued stimuli (CR) during the retention phase. Both freezing behavior and post‐mortem measurement of network activation (c‐fos expression) were used to demonstrate the difference between MS time patterns in artificially created neuronal representations of the learned task.

## 2 Methods

### 2.1 Animals and Experimental Design

Adult male C57BL/6J mice (8–12 weeks old) were used in this study. The animals were obtained from the Central Animal Facility of the Universidade Federal de Minas Gerais (UFMG) and housed in the mouse facility of the Department of Physiology and Biophysics, Institute of Biological Sciences, UFMG. Mice were maintained in standard polypropylene cages (30 × 20 × 13 cm; maximum of five animals per cage) under controlled environmental conditions (temperature: 22°C ± 1°C; humidity: 40%–70%; 12:12 h light/dark cycle). Food and water were provided ad libitum. All procedures were approved by the Institutional Animal Care and Use Committee (CEUA‐UFMG; protocol number 339/2023) and conducted in compliance with the guidelines for the care and use of laboratory animals.

### 2.2 Stereotaxic Placement of Stimulation Electrodes

Mice were anesthetized with a gaseous mixture of isoflurane (0.5%–0.8%) in oxygen, and the scalp area was locally anesthetized with Lidocaine Hydrochloride before stereotaxic surgery (Stereotaxic apparatus: Stoelting Co., Wood Dale, IL, USA). After confirming the absence of reflex responses, the scalp was shaved and disinfected with 10% povidone‐iodine and 70% ethanol. The skull was exposed, and the periosteum was removed to locate the bregma and lambda landmarks. Bipolar electrodes (0.5 mm tip separation) were created from twisted pairs of Teflon‐coated stainless‐steel wires (Model 791400, A‐M Systems Inc., Carlsborg, WA, USA) and implanted bilaterally into the CA3 region of the hippocampus at the following coordinates: anteroposterior (AP): −2.90 mm; mediolateral (ML): ±3.2 mm; dorsoventral (DV): −3.2 mm, based on (Paxinos and Franklin 2001). Although electrodes were implanted bilaterally, stimulation was delivered exclusively to the left hemisphere to avoid confounding effects of mechanical lesions on neuronal activation.

Electrodes were secured to the skull using zinc‐based dental cement and connected to a commercial 18‐pin nano dual‐row female connector (Omnetics, Minneapolis, MN, USA), which was fixed in place with acrylic resin. Following surgery, animals received a single subcutaneous injection of a pentabiotic solution (19 mg/kg) and flunixin meglumine (5 mg/kg) for prophylaxis against infection and anti‐inflammatory support. A post‐operative recovery period of 5–7 days was allowed before experimental procedures began.

### 2.3 Deep‐Brain Hippocampal Stimulation Protocol

**FIGURE 1:**
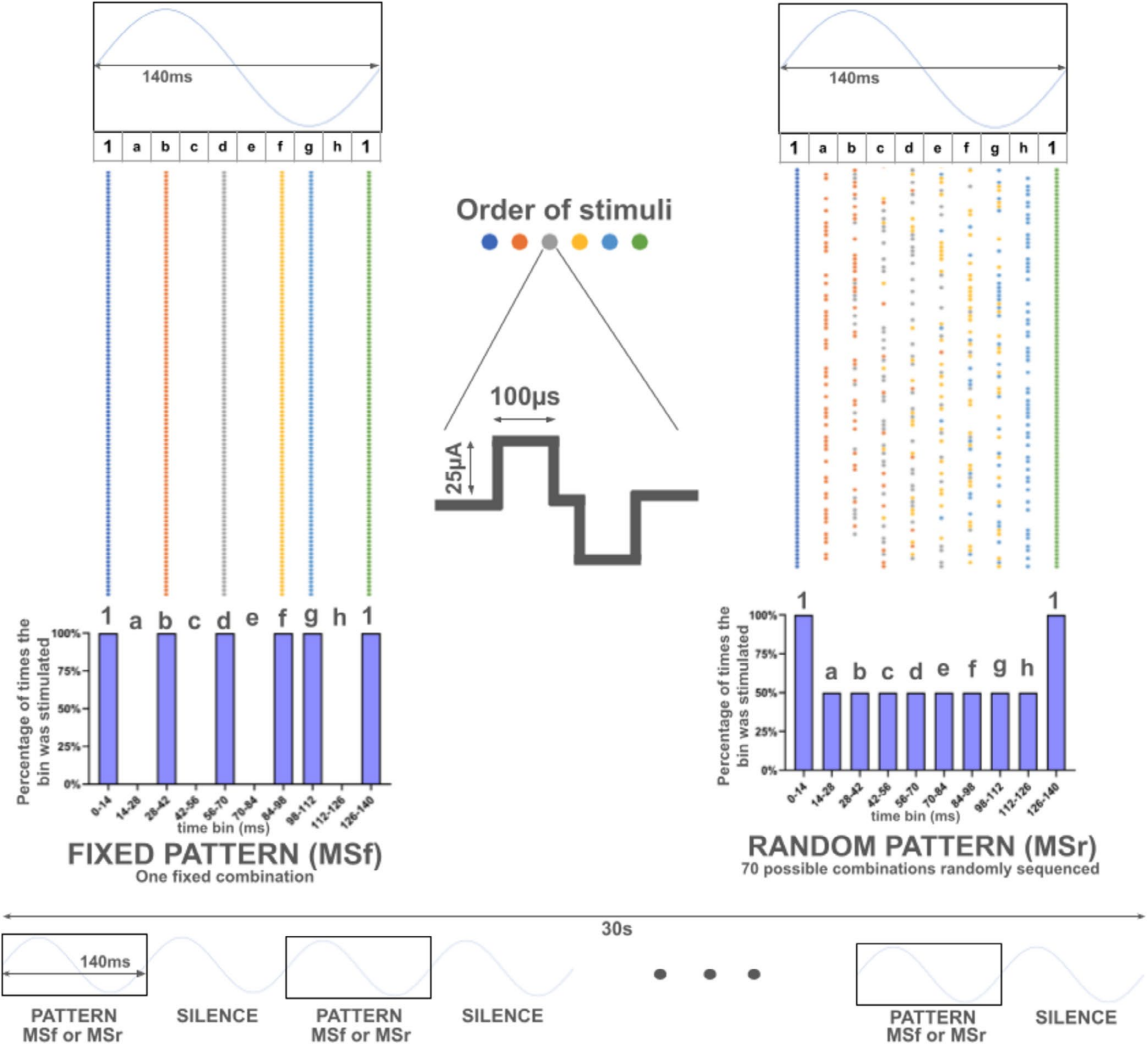
Schematic representation of the two intra‐hippocampal phase‐encoding temporal microstimulation patterns employed in the study: MSf (Fixed MicroStimulation pattern) and MSr (Random MicroStimulation pattern). In both paradigms, micro‐electrical stimuli (25 μA/100 μs biphasic square pulses) were delivered within a 140 ms cycle (~7 Hz), shown as 10‐bit binary‐coded words, where each bit represents a 14‐ms time bin. For both MSf and MSr, every word begins and ends with a stimulus pulse (starting with blue and ending with green). Each of the six dots indicates the bin in which a stimulus occurred. Histograms of stimuli occurrence percentage per bin in the complete 30s training task for both MSf (left) and MSr (right) patterns. The MSf delivers the same word repeatedly, maintaining consistent phase relationships (although not constant interpulse intervals) within the artificially generated theta period. Conversely, the MSr delivers a random sequence of 70 possible combinations of stimulus words within the theta‐period. Each group received only one temporal pattern (i.e., MSf or MSr). The patterns were applied during the 30s presentation of a pure tone (CS), as shown in the bottom panel, that terminated with a 2 s/0.4 mA footshock (US).

Hippocampal microstimulation (MS) consisted of biphasic square‐wave pulses (100 μs, 25 μA—Figure 1 waveform inset) delivered by a constant‐current stimulator (Digitimer DS2A‐Mk). Pulse timing was controlled through TTL (transistor‐transistor logic) signals generated by an Atmel SAM3X8E ARM Cortex‐M3 microcontroller, which was programmed using the ARDUINO DUE platform. Stimulation patterns were designed to encode temporally structured signals framed within an averaged theta cycle (~140 ms, ~7 Hz), with each stimulus represented as a 10‐bit word, where each bit corresponded to a 14‐ms bin. Within these binary‐coded words, “1” indicated a stimulation pulse and “0” indicated no stimulation. All words began and ended with a stimulus pulse and were followed by a theta‐length pause to prevent overlap between consecutive words (Figure 1).

Two stimulation protocols were employed (see Figure 1): (i) a FIXED pattern, in which the same word was repeated throughout the session, and (ii) a RANDOM pattern, where a different word was presented at each framed theta cycle. In both conditions, the identical hippocampal target site received six pulses per 140 ms window, all starting and finishing the theta period with a stimulus, differing only in the temporal distribution (phase) of the pulses across the inner theta cycle. Stimulation patterns and system schematics are available at: https://github.com/nnc‐ufmg/nnc_projects/tree/main/STIMULATOR%20TRIGGER.

### 2.4 Cued Fear Conditioning Protocol

The fear conditioning protocol was conducted using a custom‐built chamber developed in‐house (Amaral‐Júnior et al. 2019) and placed within a sound‐attenuating enclosure (530 × 650 × 500 mm, Bonther). The chamber featured a stainless‐steel grid floor composed of 16 parallel cylindrical bars (250 mm long, 5 mm in diameter, spaced 20 mm apart), which were connected to an electrical stimulator for delivering footshocks. The chamber walls were constructed from acrylic, allowing for interchangeable colored inserts to create distinct contextual environments. Auditory stimuli were generated via a digital‐to‐analog converter using the Arduino Due platform and delivered through a speaker mounted on the chamber (Amaral‐Júnior et al. 2019). Animal behavior was monitored with a top‐mounted camera (Microsoft LifeCam VX‐700).

Fear conditioning took place during the animals' light phase. Mice were exposed to two distinct contexts: Context A, designated for habituation and testing, and Context B, utilized for training, which were distinguished by visual, olfactory, and tactile cues. Context A had transparent acrylic walls, a floor covered with a textured surface to deter contact with the metal bars, and was scented with a 1% acetic acid solution. Context B featured four vertically striped black‐and‐white panels and an exposed grid floor, and was scented with 70% ethanol. Both contexts were acoustically isolated.

Before the experiment, all animals underwent daily handling sessions of 10 min each for 7 consecutive days. Mice were randomly assigned to one of three groups: FIXED Pattern—received electrical microstimulation using fixed binary‐coded temporal sequences; RANDOM Pattern—received microstimulation with randomized binary‐coded sequences; and Sham—connected to the stimulation system but did not receive any electrical stimulation.

The auditory fear conditioning protocol consisted of three phases: habituation, training, and testing, conducted over 3 consecutive days. During habituation, the animals were connected to the stimulation setup and placed in Context A for 10 min of free exploration. A paper towel soaked with 70% ethanol was placed beneath the chamber drawer to establish olfactory cues. No stimuli were presented during this phase.

Twenty‐four hours later, the training session took place in Context B. The stimulator parameters (pulse amplitude, frequency, and waveform) were verified using an oscilloscope before each session. Animals were connected to the stimulation system and positioned in the center of the chamber for 120 s of free exploration. This was followed by a 30‐s presentation of a 1 kHz pure tone (conditioned stimulus, CS), which was temporally paired with electrical microstimulation. In the final 2 s of the CS, a footshock (0.4 mA, unconditioned stimulus, US) was delivered concurrently with the tone and microstimulation. Only one CS‐US pairing was administered. After the pairing, animals remained in the chamber for 30 s before being returned to their home cages.

The test session occurred 24 h after training. Mice were re‐exposed to Context A. A paper towel soaked in 70% ethanol was again placed under the chamber drawer. The animals were allowed 120 s of free exploration, followed by a 30‐s presentation of the CS. After the tone offset, the animals remained in the chamber for an additional 30 s before removal.

Conditioned freezing behavior—defined as the absence of all movement except for respiration for at least 3 s within a 5‐s interval (Blanchard and Blanchard 1972, 1969; Bouton and Bolles 1980; Fanselow and Bolles 1979)—was quantified from video recordings by an observer blind to experimental conditions. Freezing was analyzed across habituation and test phases in three distinct epochs: pre‐tone, tone, and post‐tone. Data were expressed as the percentage of time spent freezing in each epoch.

### 2.5 Tissue Processing and Histological Verification

At the end of the experimental procedures, the animals were deeply anesthetized with an intraperitoneal injection of ketamine (80 mg/kg) and xylazine (30 mg/kg). An electrolytic lesion was performed (0.5 mA for 2 s) to confirm the position of the implanted electrodes. Subsequently, the animals were transcardially perfused with 10 mL of phosphate‐buffered saline (PBS; 0.1 M, pH 7.4; containing 0.387 M NaH_2_PO_4_·H_2_O, 0.612 M Na_2_HPO_4_·7H_2_O, and 1.4 M NaCl), followed by 10 mL of a 4% paraformaldehyde (PFA) solution in PBS (w/v, pH 7.4).

The brains were carefully removed and post‐fixed in 4% PFA at 4°C for 24 h. After fixation, they were cryoprotected by immersion in a 30% sucrose solution (w/v in PBS) until fully saturated. Coronal sections (40 μm thick) were obtained using a cryostat (Leica or MC‐00, LUPE Indústria e Comércio) and stored in a cryoprotective solution containing 30% sucrose (w/v), 30% ethylene glycol (v/v), 1% polyvinylpyrrolidone (w/v), and 0.1 M PBS, at −20°C until further immunofluorescence processing.

Brain sections from the prefrontal cortex, amygdala complex, and hippocampus were collected for immunofluorescence analysis. To verify electrode and cannula placement, only hippocampal sections were selected and stained with a neutral red solution (1% neutral red w/v, 0.3% anhydrous sodium acetate w/v, 0.12% glacial acetic acid v/v), based on stereotaxic coordinates corresponding to the implantation sites.

### 2.6 Immunofluorescence and Image Analysis

After the cued fear conditioning paradigm—specifically, 90 min after the final conditioned stimulus (CS) presentation on the third day of the retention test—all animals were sacrificed and transcardially perfused as described in Section 2.5. This time point was chosen to capture the peak expression of c‐Fos protein (Knapska and Maren 2009; Worley et al. 1993). Brains were sectioned and stored in an antifreeze solution before immunofluorescence processing. A triple‐labeling protocol was employed to assess neuronal activation using antibodies against c‐Fos (Miller et al. 1984), NeuN (Mullen et al. 1992), and the nuclear marker DAPI (Russell et al. 1975).

Free‐floating coronal sections were initially rinsed three times for 5 min each in phosphate buffer (PB 0.1 M, pH 7.4). This was followed by three washes in PBS‐T (PBS 0.1 M + 0.3% Triton X‐100, v/v). Sections were then incubated for 1 h at room temperature in a blocking solution containing PB 0.1 M and 3% bovine serum albumin (BSA). After blocking, the sections were incubated overnight (16 h, room temperature) in a solution containing the primary antibodies: rabbit anti‐c‐Fos (1:1000; Santa Cruz Biotechnology) and mouse anti‐NeuN (1:2000; Millipore) diluted in PBS‐T with 3% BSA.

Following primary incubation, sections were rinsed three times in PB and then incubated for 2 h at room temperature (protected from light) with the secondary antibodies: Alexa Fluor 555 goat anti‐rabbit (1:1000; Molecular Probes) and Alexa Fluor 647 goat anti‐mouse (1:2000; Molecular Probes), also diluted in PBS‐T with 3% BSA. Subsequently, sections were incubated with DAPI (1:10,000; Molecular Probes) for 5 min and rinsed in PB. Slices were mounted onto Superfrost Plus slides using Fluoromount aqueous mounting medium, protected from light, and stored at 4°C until analysis.

Immunoreactivity (IR) for c‐Fos was quantified in defined regions of interest (ROIs), specifically the amygdaloid complex—including the lateral (LA), central (Ce), and basolateral (BLAe) nuclei—and the medial prefrontal cortex (mPFC), comprising the prelimbic (PrL) and infralimbic (IL) cortices (Marek et al. 2013). ROIs were defined according to stereotaxic coordinates from Paxinos and Franklin (2001) [AP: +3.20 mm to +2.20 mm] (Paxinos and Franklin 2001). Images were acquired using a Zeiss Axio Imager.M2 fluorescence microscope with 5×, 20×/0.5 NA, and 100×/1.3 NA objectives and analyzed using Axiovision 4.8 software.

Three to five images were captured and averaged for each region of interest (ROI) to obtain a representative value. Quantification was performed on images acquired at 20× magnification under consistent exposure settings across all samples. Z‐stacks (≥ 4 μm optical depth) were collected using filter sets appropriate for each fluorophore (c‐Fos: 450–490/515 nm; NeuN: 640/690 nm; DAPI: 365/445 nm). Raw image files were processed and analyzed in ImageJ (http://rsbweb.nih.gov/ij/). The autofluorescence background was subtracted using a fixed threshold applied uniformly across all images. Colocalization of c‐Fos and NeuN was assessed using the Colocalization Threshold plugin (http://fiji.sc/Colocalization_Threshold) (Costes et al. 2004).

Colocalized cells were converted to 8‐bit images, and objects with areas between 30 and 40 μm^2^ were counted as IR‐positive cells. Total NeuN‐positive neurons were identified by colocalization with DAPI and quantified using the same criteria. The final values were expressed as the percentage of c‐Fos+/NeuN+ cells relative to the total number of NeuN+ neurons in each ROI. The final images were digitally reconstructed and overlaid for visualization.

### 2.7 Statistical Analysis

Data distribution was first assessed for normality using the Kolmogorov–Smirnov test. As the assumption of normality was met, parametric statistical tests were applied.

Behavioral data from the auditory fear conditioning paradigm, expressed as the percentage of freezing, were analyzed using one‐way or two‐way analysis of variance (ANOVA), depending on the experimental design. When freezing responses were evaluated across different temporal phases within the same subjects (pre‐tone, tone, and post‐tone periods), a two‐way repeated‐measures ANOVA was performed, with experimental group (Control, Organized pattern, Random pattern) as the between‐subjects factor and test phase as the within‐subjects factor; post hoc multiple comparisons were conducted using Bonferroni's or Dunnett's correction, as appropriate, to control for multiple testing.

For immunohistochemical analyses, the number of c‐Fos–positive cells per area in each region of interest was analyzed using one‐way ANOVA, with the experimental group (Naive, Control, Organized pattern, Random pattern) as the independent factor. Post hoc tests were applied when necessary to identify group‐specific differences.

Associations between behavioral performance (freezing percentage) and neuronal activation (c‐Fos immunoreactivity) were examined using Pearson's correlation coefficient.

All data are presented as mean ± SEM. Statistical significance was set at *p* < 0.05. All analyses were performed using GraphPad Prism (version 9.5).

## 3 Results

### 3.1 Behavioral Freezing Response

According to the results, the application of the Fixed Pattern of electrical microstimulation (EM) as the conditioned stimulus met our expectations. Only during the test, in the 30‐s period in which the sound (CS) was presented, EM was able to elicit a conditioned fear response (freezing) in the group that received the temporally organized stimulus during the training phase—when the CS‐US association was established. During the pre‐tone phase, a two‐way repeated‐measures ANOVA followed by Bonferroni's multiple‐comparisons test revealed no significant differences among groups, indicating comparable baseline behavior (Sham vs. Fixed: mean difference = −7.83%, 95% CI [−16.89, 1.23], *p* = 0.0966; Sham vs. Random: mean difference = −10.26%, 95% CI [−26.13, 5.61], *p* = 0.2274; Fixed vs. Random: mean difference = −2.43%, 95% CI [−18.39, 13.52], *p* > 0.9999). During tone (CS) presentation, freezing behavior differed between groups. Animals receiving the fixed stimulation pattern exhibited a significantly higher freezing response (69.33% ± 5.01%) compared with the Sham group (mean difference = −28.23%, 95% CI [−55.42, −1.03], *p* = 0.0412). No significant differences were observed between the Sahm and Random groups (mean difference = −9.93%, 95% CI [−53.12, 33.27], *p* > 0.9999) or between the Fixed and Random groups (mean difference = 18.30%, 95% CI [−24.49, 61.09], *p* = 0.6959).

During the post‐tone phase, freezing responses did not differ among groups (Sham vs. Fixed: mean difference = −19.11%, 95% CI [−50.03, 11.81], *p* = 0.3206; Sahm vs. Random: mean difference = −1.79%, 95% CI [−25.75, 22.17], *p* > 0.9999; Fixed vs. Random: mean difference = 17.33%, 95% CI [−15.30, 49.95], *p* = 0.4928). These findings suggest that the Fixed Pattern of electrical stimulation was capable of influencing the animals' behavior during the CS. In contrast, the Random Pattern had no significant effect compared to the Sham.

### 3.2 Temporally Organized Stimulation Increases Amygdala Neuronal Activation

**FIGURE 2:**
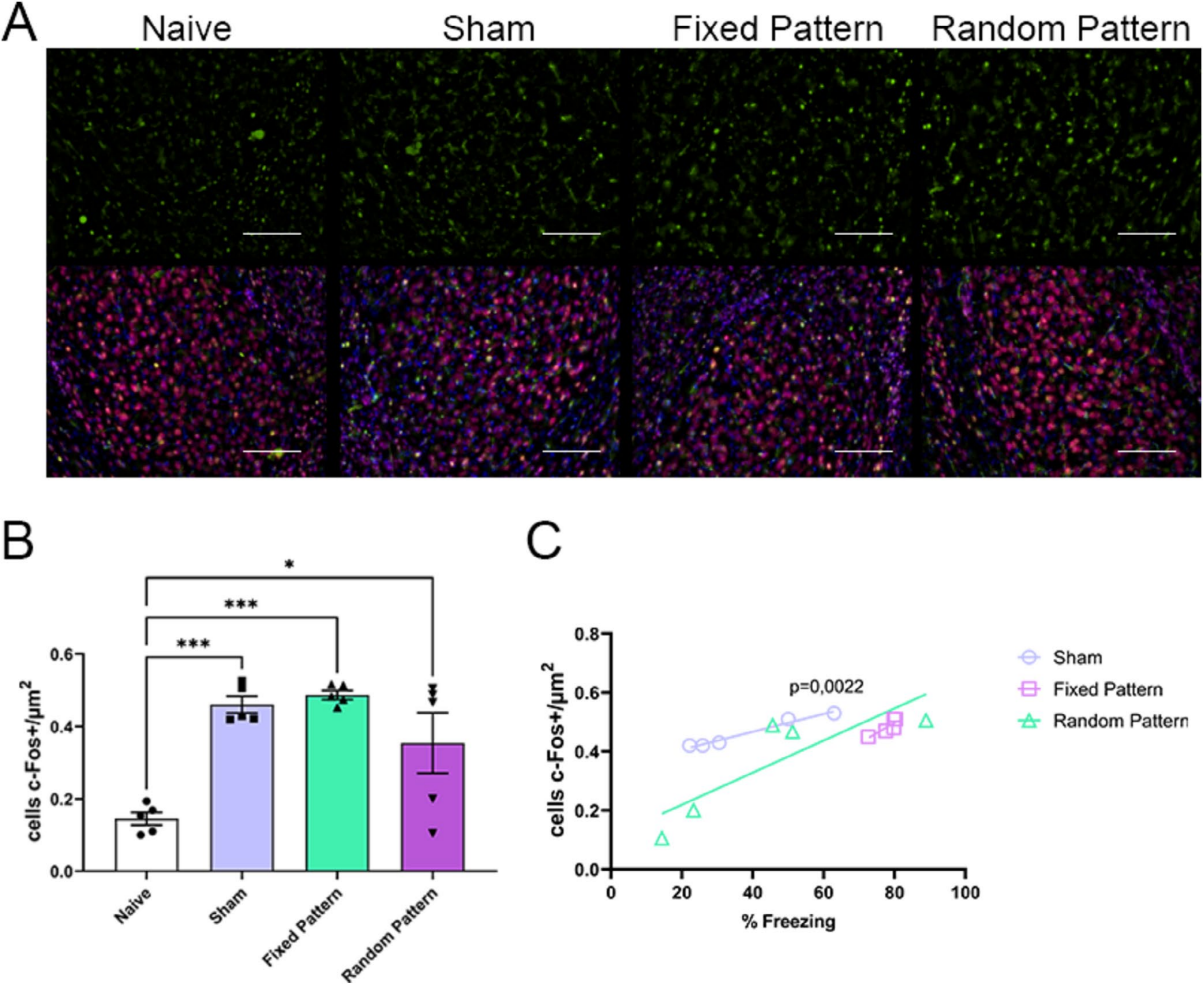
c‐Fos expression in the amygdala across experimental groups. (A) Representative images of c‐Fos immunoreactivity (green, top) and merged labeling with NeuN (red) and DAPI (blue, bottom) in the amygdala of Naive, Sham, Fixed Pattern and Random Pattern groups. Scale bar = 100 μm. (B) Quantification of c‐Fos + cells (cells/μm^2^) in the amygdala. All experimental groups (Sham, Fixed Pattern, and Random Pattern) showed significantly increased c‐Fos expression compared to Naive. (C) Correlation between freezing behavior and c‐Fos expression in the amygdala, with a significant positive association observed in the Sham group (*p* = 0.0022). Data are presented as mean ± SEM. One‐way ANOVA followed by Tukey's post hoc test: *p < 0.05; **p < 0.01; ***p < 0.001.

Quantification of c‐Fos–positive neurons in the amygdala (AMY) revealed a significant effect of experimental group. One‐way ANOVA showed a robust group effect (*F*(3,16) = 12.11, *p* = 0.0002, *R* ^2^ = 0.69). Post hoc analyses indicated that the Fixed pattern group exhibited significantly higher c‐Fos expression compared with both the Sham and Random groups (*p* < 0.05), whereas no difference was observed between Sham and Random groups (*p* > 0.05) (Figure 2B).

### 3.3 Differential Recruitment of Dorsal Hippocampal Subregions

**FIGURE 3:**
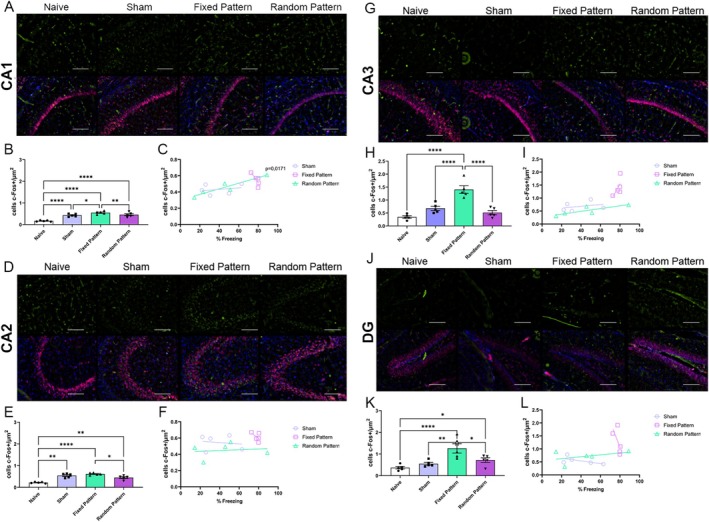
Dorsal hippocampal c‐Fos expression across experimental groups. (A, D, G, J) Representative photomicrographs illustrating c‐Fos immunoreactivity (green, upper panels) and merged images with NeuN (red) and DAPI (blue) in the dorsal hippocampus, encompassing CA1 (A), CA2 (D), CA3 (G), and dentate gyrus (DG; J), across Naive, Sham, Fixed Pattern, and Random Pattern groups. Scale bars = 100 μm. (B, E, H, K) Quantification of c‐Fos–positive cells (cells/μm^2^) in dorsal CA1 (B), CA2 (E), CA3 (H), and DG (K). Relative to Naive animals, both Sham and Fixed Pattern groups exhibited increased c‐Fos expression across regions, with the Fixed Pattern group showing the strongest activation particularly in CA3 and DG. The Random Pattern group displayed intermediate or region‐specific activation levels. (C, F, I, L) Correlation analyses between freezing behavior (%) and c‐Fos expression in CA1 (C), CA2 (F), CA3 (I), and DG (L) for Sham, Fixed Pattern, and Random Pattern groups. A significant positive correlation between freezing and c‐Fos density was observed exclusively in CA1 for the Random Pattern group (*p* = 0.0171), while no significant correlations were detected in the other regions or groups. Data are presented as mean ± SEM. Statistical analyses were performed using one‐way ANOVA followed by Tukey's post hoc test. Significance levels are indicated as *p < 0.05, **p < 0.01, and ****p < 0.0001.

In the dorsal hippocampus, robust region‐specific effects of experimental condition were observed. In dorsal CA1, one‐way ANOVA revealed a significant group effect (*F*(3,16) = 25.24, *p* < 0.0001, *R* ^2^ = 0.83), with increased c‐Fos expression in the Fixed group relative to Sham and Random groups (*p* < 0.05) (Figure 3B). Similarly, dorsal CA2 showed a significant group effect (*F*(3,16) = 27.84, *p* < 0.0001, *R* ^2^ = 0.84), with higher neuronal activation in the Fixed group compared with Sham and Random groups (*p* < 0.05) (Figure 3E). In dorsal CA3, one‐way ANOVA indicated a strong effect of group (*F*(3,16) = 23.23, *p* < 0.0001, *R* ^2^ = 0.81). Fixed stimulation produced significantly greater c‐Fos expression compared with Sham and Random conditions (*p* < 0.05) (Figure 3H). In the dorsal dentate gyrus, group differences were also detected (*F*(3,16) = 9.08, *p* = 0.0010, *R* ^2^ = 0.63), with increased neuronal activation in the Fixed group relative to Sham and Random groups (*p* < 0.05) (Figure 3K).

### 3.4 Recruitment of Ventral Hippocampal Circuits

**FIGURE 4:**
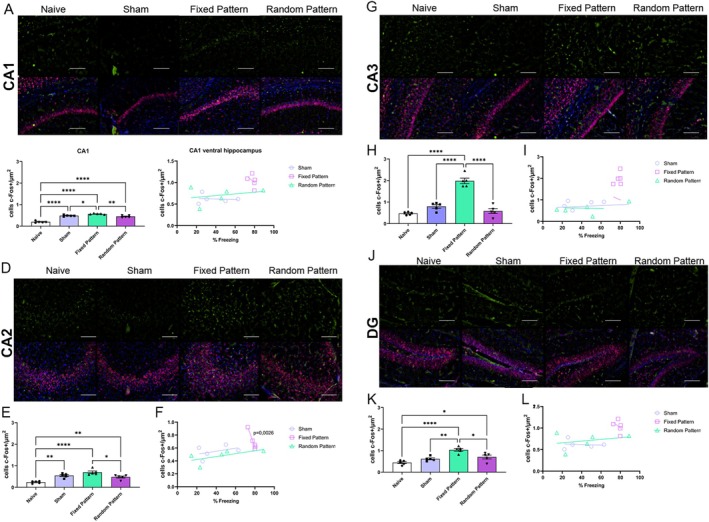
c‐Fos expression in the ventral hippocampus across experimental groups. (A, D, G, J) Representative photomicrographs showing c‐Fos immunoreactivity (green, upper panels) and merged images with NeuN (red) and DAPI (blue, lower panels) in the ventral hippocampus, including CA1 (A), CA2 (D), CA3 (G), and dentate gyrus (DG; J), from Naive, Sham, Fixed Pattern, and Random Pattern groups. Scale bars = 100 μm. (B, E, H, K) Quantification of c‐Fos–positive cells (cells/μm^2^) in ventral CA1 (B), CA2 (E), CA3 (H), and DG (K). All experimental groups exhibited significantly higher c‐Fos expression compared to Naive animals, with region‐specific differences among Sham, Fixed Pattern, and Random Pattern conditions. (C, F, I, L) Correlation analyses between freezing behavior (%) and c‐Fos expression in ventral CA1 (C), CA2 (F), CA3 (I), and DG (L). A significant positive correlation between freezing behavior and c‐Fos density was observed exclusively in ventral CA2 for the Fixed Pattern group (*p* = 0.0026), whereas no significant associations were detected in the other subfields or experimental groups. Data are presented as mean ± SEM. Statistical analyses were performed using one‐way ANOVA followed by Tukey's post hoc test. Significance levels are indicated as *p < 0.05, **p < 0.01, and ****p < 0.0001.

Analysis of the ventral hippocampus revealed a consistent enhancement of neuronal activation associated with Fixed stimulation. In ventral CA1, one‐way ANOVA demonstrated a significant group effect (*F*(3,16) = 25.24, *p* < 0.0001, *R* ^2^ = 0.83), with higher c‐Fos expression in the Fixed group compared with Sham and Random groups (*p* < 0.05) (Figure 4B). Similarly, ventral CA2 showed a significant main effect of group (*F*(3,16) = 27.84, *p* < 0.0001, *R* ^2^ = 0.84), with the Fixed group exhibiting greater neuronal activation than Sham and Random groups (*p* < 0.05) (Figure 4E). In ventral CA3, the stimulation target region, one‐way ANOVA revealed a very strong group effect (*F*(3,16) = 68.02, *p* < 0.0001, *R* ^2^ = 0.93). Animals exposed to the Fixed stimulation pattern displayed markedly increased c‐Fos expression compared with Sham and Random groups (*p* < 0.05) (Figure 4H). In the ventral dentate gyrus, group differences were also observed (*F*(3,16) = 9.08, *p* = 0.0010, *R* ^2^ = 0.63), again reflecting enhanced neuronal recruitment in the Fixed group (*p* < 0.05) (Figure 4K).

### 3.5 Prefrontal Cortex Activation Is Region Specific

**FIGURE 5:**
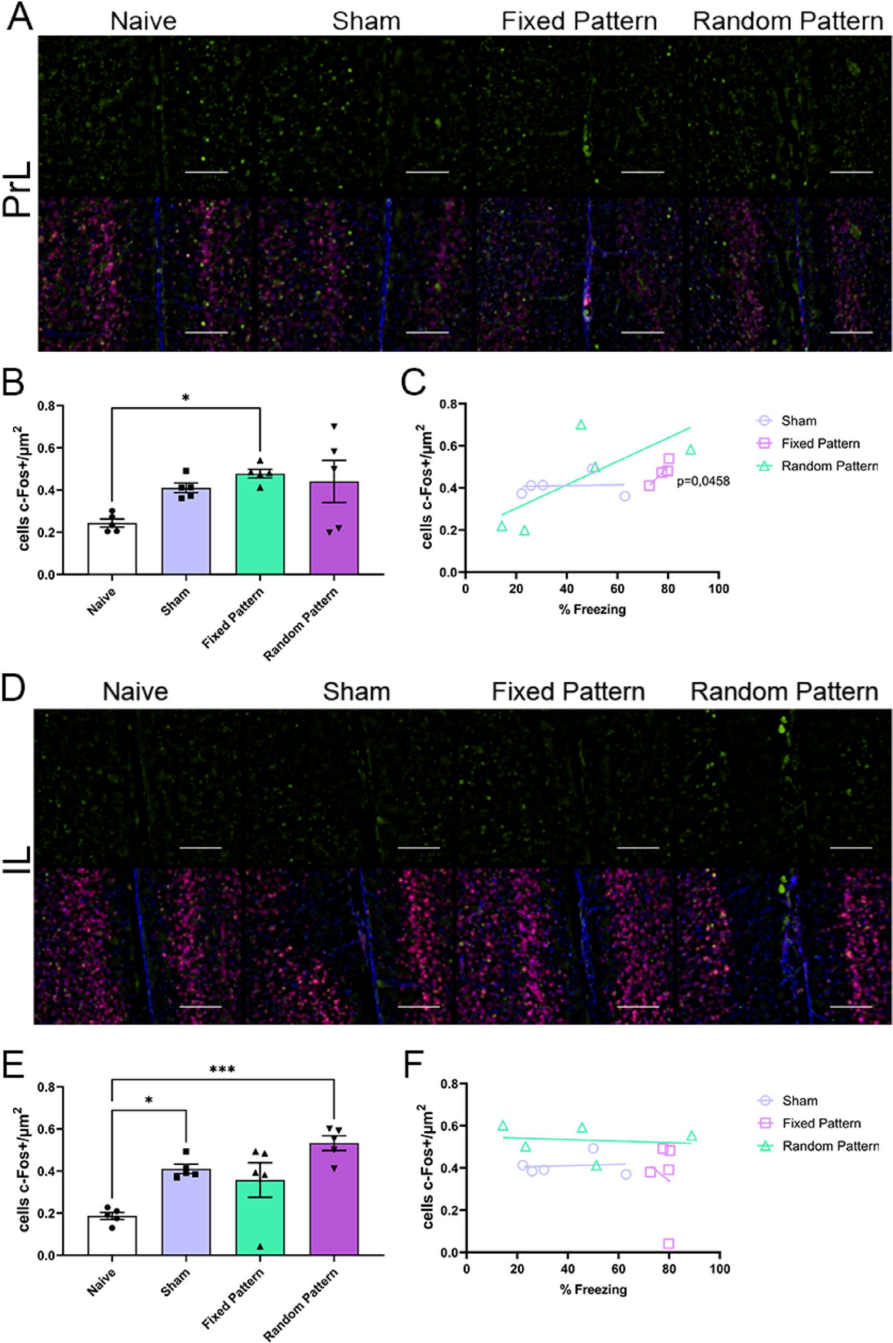
c‐Fos expression in the medial prefrontal cortex across experimental groups. (A) Representative images of c‐Fos immunoreactivity (green, top) and merged labeling with NeuN (red) and DAPI (blue, bottom) in the prelimbic cortex (PrL). Scale bar = 100 μm. (B) Quantification of c‐Fos + cells (cells/μm^2^) in PrL. Only Fixed Pattern showed higher c‐Fos expression compared to Naive. (C) Correlation between freezing behavior and c‐Fos expression in PrL, showing a significant positive association for the Fixed Pattern group (*p* = 0.0458). (D) Representative images of c‐Fos immunoreactivity (green, top) and merged labeling with NeuN (red) and DAPI (blue, bottom) in the infralimbic cortex (IL). Scale bar = 100 μm. (E) Quantification of c‐Fos + cells in IL. Sham and Random Pattern groups showed significantly higher c‐Fos expression compared to Naive. (F) Correlation between freezing and c‐Fos expression in IL, with no significant associations. Data are presented as mean ± SEM. One‐way ANOVA followed by Tukey's post hoc test: *p < 0.05; ***p < 0.001.

In the prelimbic cortex, one‐way ANOVA revealed a significant group effect (*F*(3,16) = 3.80, *p* = 0.0313, *R* ^2^ = 0.42), with increased c‐Fos expression in the Fixed group compared with Sham and Random groups (*p* < 0.05) (Figure 5B). In contrast, in the infralimbic cortex, although a significant main effect of group was detected (*F*(3,16) = 9.42, *p* = 0.0008, *R* ^2^ = 0.64), post hoc analyses did not support a consistent increase specifically associated with the Fixed pattern, indicating a distinct recruitment profile relative to the prelimbic cortex (Figure 5E).

### 3.6 Correlation Between Neuronal Activation and Conditioned Freezing Behavior

Pearson correlation analyses were performed to examine the relationship between freezing behavior during tone presentation and c‐Fos immunoreactivity across amygdala, hippocampal, and prefrontal regions.

A strong positive correlation was observed between freezing and amygdala c‐Fos expression in the Sham condition (*r* = 0.9851, 95% CI [0.7859, 0.9991], *R* ^2^ = 0.9704, *p* = 0.0022). No significant correlations were detected for the Fixed or Random conditions (*p* = 0.0584 and *p* = 0.0695, respectively) (Figure 2C).

In the dorsal hippocampus, a significant positive correlation was detected between freezing and dorsal CA1 c‐Fos expression specifically in the Random condition (*r* = 0.9408, 95% CI [0.3446, 0.9962], *R* ^2^ = 0.8851, *p* = 0.0171), whereas correlations in Sham and Fixed conditions were not significant (*p* = 0.6884 and *p* = 0.1050, respectively) (Figure 3C). No significant correlations were found for dorsal CA2, dorsal CA3, or dorsal dentate gyrus across Sham, Fixed, or Random conditions (all *p* > 0.05) (Figure 3F,I,L).

In the ventral hippocampus, no significant correlations were observed for ventral CA1, ventral CA3, or ventral dentate gyrus in any condition (all *p* > 0.05) (Figure 4C,I,L). However, a strong negative correlation was detected between freezing and ventral CA2 c‐Fos expression in the Fixed pattern condition (*r* = −0.9834, 95% CI [−0.9990, −0.7637], *R* ^2^ = 0.9670, *p* = 0.0026), while Sham and Random conditions were not significant (*p* = 0.4848 and *p* = 0.2534) (Figure 4F).

In the prelimbic cortex (PrL), freezing correlated positively with c‐Fos expression in the Fixed pattern condition (*r* = 0.8854, 95% CI [0.0143, 0.9924], *R* ^2^ = 0.7839, *p* = 0.0458), but not in Sham or Random conditions (*p* = 0.9054 and *p* = 0.1652) (Figure 5C). In the infralimbic cortex (IL), no significant correlations were observed in any condition (all *p* > 0.05) (Figure 5F).

## 4 Discussion

**FIGURE 6:**
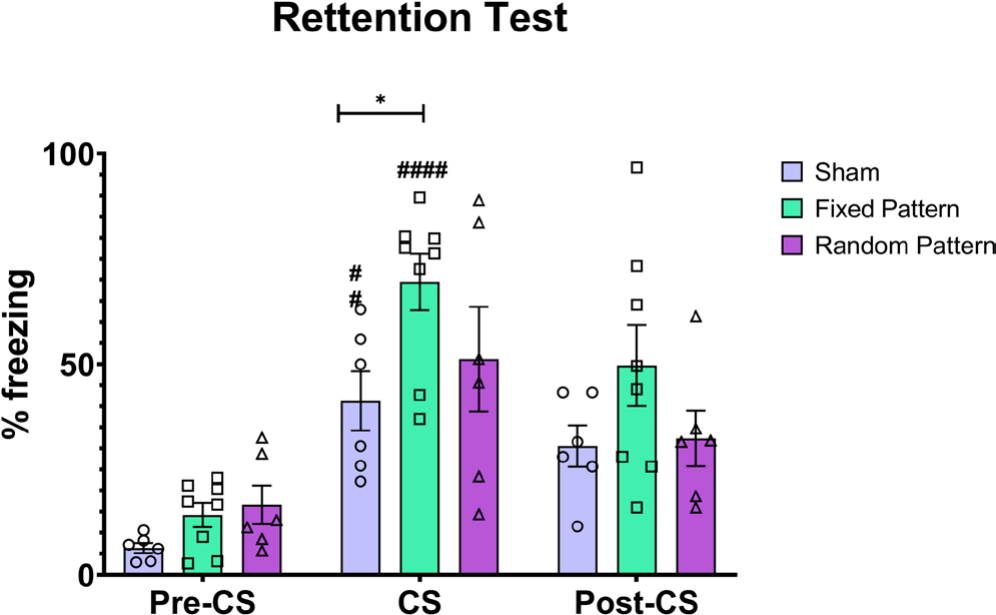
Behavioral freezing response in the Sham, Fixed Pattern, and Random Pattern groups during the Pre‐CS, CS, and Post‐CS periods in the auditory fear conditioning test. Data are presented as mean ± standard error of the mean (SEM). The symbol ## indicates statistically significant differences compared to the Pre‐CS period (*p* < 0.01), whereas #### indicates differences with *p* < 0.0001. The symbol * indicates significant differences between the Sham and Organized groups within the same period (*p* < 0.05).

Our results show that MS applied to the hippocampus, at the same loci and with the same overall number of stimuli per averaged theta period, differing only in the phase organization framed within the averaged 140‐ms theta period, is determinant for hippocampal recruitment during a hippocampus‐independent classical cued fear conditioning response. Moreover, since all patterns (both MSr and MSf) start and finish, within its 140 ms period, with a stimulus; the authors argue that it is unlikely that the observed effect is due to Phase‐Resetting alone. Also, the MSf pattern intentionally did not have evenly spaced interpulse intervals within the theta‐stimulation period; it was still a more potent stimulus to drive hippocampal circuit involvement than Sham; however, MSr did not differ from Sham. Evidence suggests that not only was the behavioral CR potentiated by the Fixed MS pattern (Figure 6 showing increased freezing behavior in the Fixed group), but also that c‐fos activation in several hippocampal structures, due to CS applied during the retrieval phase, was higher for the Fixed MS pattern (Figures 3 and 4). Altogether, this data suggests that temporal coding (neurostimulation based on a theta phase‐encoding cycle) is a potent parameter to drive hippocampal neuronal representation and trace memory formation. In other words, the plastic changes necessary for the CS to evoke a higher activation of hippocampal neuronal ensembles during retrieval only occurred when, during training, CS was paired to the US and the MS was temporally organized in phases framed within a 140 ms period; and, considering the patterns used, may involve other mechanisms than single‐pulse phase‐resetting alone. Nevertheless, Amygdaloid Complex neuronal activation was equally present with or without hippocampal involvement in sham, Fixed MS, and Randomized MS groups (Figure 2), suggesting circuits underlying the hippocampus‐independent cued response were present in all groups. The authors argue in the following paragraphs that the Fixed MS patterns may have created artificial context‐like engrams in the hippocampus.

If, as hypothesized, a specific word composed of 10 different hippocampal‐theta phase bins would favor one specific circuit activation (i.e., engram or special trace representation within the neuronal network ensemble), it could, if repeated, consequently induce plastic changes to that circuit in a very precise time‐dependent manner, compatible with STDP (Basu et al. 2016; Feldman 2012). Conversely, a randomized word would recruit several competing circuits, not favoring any particular one during training, thus not causing plastic changes. This hypothesis can only be properly addressed with concomitant recordings that evaluate real‐time neurodynamic such as electrophysiological, miniscope (GCaMP calcium imaging) or fiber photometry recordings; these are examples of ongoing experiments in our laboratory. In the Lipponen et al. artificially restored theta rhythm oscillation experiment (Lipponen et al. 2012), sham‐operated animals subjected to stimulation also exhibited impaired contextual conditioned responses; perhaps, according to the proposed rationale, because the stimulation itself acted as a competing circuit. Accordingly, the stimulation in this case was not, as intended, an artificially induced theta oscillation, but rather a competing word/engram in the hippocampal memory trace representation of the context. Previous work from our group supports the idea that a fixed pattern of MS favors one specific circuit, creating a “winner‐takes‐all” dynamic process (Chen 2017; Kim and Lim 2022; Rutishauser et al. 2011; Winder 1999). In contrast, the corollary argument for this explanation, a random pattern would evoke multiple competing circuits, all struggling for system control, making it difficult for any specific circuit to dominate the network behavior. Using animal models of epilepsy, we have shown that a Fixed pattern is proconvulsive while a Random pattern is anti‐convulsant (Cota et al. 2009, 2023). In a sequence of fMRI images of animals subjected to an i.v. ramp of a proconvulsive substance (Pentylenetetrazol—PTZ), Mesquita et al. (2011) showed that although both patterns equally activated the stimulation site, the Fixed pattern enhanced ipsilateral activation, while the randomized pattern inhibited it. Mimicking the stimulation pattern of this work, it is important to highlight that these results had the same overall frequency of stimulation, applied at the same target area, but yielded antagonistic outcomes (proconvulsant vs. anticonvulsant) depending solely on the interpulse stimuli organization patterns and this has been confirmed by other experiments as well (Cota et al. 2009, 2016; Moraes et al. 2021; Mourão et al. 2016).

It is interesting to note that although MS (MSf or MSr) targeted the most posterior portion of the hippocampus (i.e., roughly midway between the dorsal and ventral hippocampus), the MSf pattern showed evidence of recruiting both the ventral and dorsal hippocampal structures, thus evidencing the spread of recruited neural substrates away from the stimulated site. The dorsal hippocampus is primarily connected to cortical regions involved in cognition, spatial processing, and navigation, including the entorhinal cortex, retrosplenial cortex, and medial prefrontal cortex (Amaral and Witter 1989; Bagot et al. 2015). On the other hand, the ventral hippocampus is connected to limbic structures, including the amygdala, hypothalamus, and prefrontal cortex, which are involved in emotions, stress, and motivation. This creates a functional ambiguity between the two areas (Ballesteros et al. 2014): (a) one specializes in spatial memory and navigation, while the other specializes in emotional memory and affective regulation; (b) one is more critically involved in encoding and recalling context‐related memories, while the other is more involved in anxiety‐related behaviors and fear conditioning. Therefore, in our case, perhaps the cued‐MS pairing modulated the emotional salience of the experience, creating a representation in ventral hippocampal structures, while the spatial aspect of the environment pattern separation (i.e., remembering that the MSf was applied during the whole training session) drove the circuit toward creating memory traces in the dorsal hippocampus. It would be interesting to redesign the experiment by eliminating the emotional salience aspect of the fear response, while retaining only the spatial separation pattern of the environment. This approach could help determine if the memory trace exclusively recruits the dorsal hippocampus. Ongoing research in our laboratory is addressing this issue using a spontaneous object exploration task with theta phase‐encoded MS representing the artificially generated context: the object‐context recognition task.

Since the initial discovery by O'Keefe and Dostrovsky (1971) that certain hippocampal neurons increase their firing patterns when the rat is in specific locations within its environment (O'Keefe and Dostrovsky 1971), it became evident that the hippocampus plays a role in representing space, not just in forming memories. Furthermore, O'Keefe and Recce (1993) discovered that place cell firing occurs at progressively earlier phases of the theta rhythm as the animal moves through the cell's place field—a phenomenon called phase precession, which is closely related to phase encoding and might govern the long‐range transference of information out of the hippocampus. These results support a movement, at the time, urging neuroscientists to reconsider Louis Lapicque's integrate‐and‐fire model (Abbott 1999) as the basis of cerebral processing architecture. The central issue of the debate was whether neurons in higher order information processing act effectively as slow temporal integrators or if the integration is fast enough that neurons essentially act as coincidence detectors, and that neuronal representations of the external world, along with engram formation, would therefore involve very precise timing of activation of specific neuronal ensembles (Basu et al. 2016; Engel and Singer 2001; Feldman 2012; Hasselmo et al. 2002; König et al. 1996; Tsien 2000). The proposed coincidence‐detection architecture, while explaining the evidence for phase encoding of cognitive and memory representations, would also address issues with the integrate‐and‐fire model, such as error propagation throughout the network, incompatibility with the known small‐world network topology of the brain, and multi‐modal information integration would take much longer than what is experimentally observed, among others (Engel and Singer 2001; König et al. 1996; Singer 1999; Uhlhaas and Singer 2006). In fact, much evidence from the last 20 years has supported the coincidence‐detection proposal, indicating that information processing in the cortex is highly dependent on time and temporal synchronization (Feldman 2012; Singer 1999). There are also arguments in favor of temporal encoding based on neural network topology, the biophysical properties of cortical neurons, and cortical dynamics. It is not our intention to transform this discussion into a review—see (Cota et al. 2023), but the approach used here is quite different from the neuronal unit recording strategy, local field potential coherence analysis, or phase‐amplitude data recorded during specific tasks found throughout the literature. Our approach was to drive an input, using MS at the entrance of the hippocampal tri‐synaptic circuit, of temporal patterns that would mimic the phase encoding proposal of information processing—a writing rather than reading approach. If our results had shown that the temporal organization of MS did not have an effect on either behavioral or c‐fos neuronal activation outcomes, the integrate‐and‐fire architecture based on slow temporal integrators would be a better model than temporal coding to explain cortical architecture and memory formation. However, that was not the case. Nevertheless, it is essential to realize that both schemes of architecture are not mutually exclusive within the brain and that the integrate‐and‐fire models most likely better represent the organization of caudal sensory input circuits and rostral motor output circuits. In contrast, the coincidence detector scheme is likely better suited for higher‐level cortical processing.

Theta oscillations occur with entorhinal input into the trisynaptic circuit, specifically the activation of CA3 Schaffer collaterals to CA1, and plasticity induced by Ca^++^ influx into pyramidal cells, being a marker of the hippocampus's involvement in several functional processes, such as learning and memory. Moreover, this rhythm is believed to be crucial for modifying synaptic weights and for the temporal coding and decoding of neuronal ensemble representation. A substantial amount of effort has been devoted to investigating the correlation between cellular‐synaptic activation in hippocampal neurons and theta wave oscillations, as well as how this process might explain the network behavior emerging from individual neurons. A key aspect is that this understanding may help clarify how the network isolates specific neuronal ensembles and redirects or coordinates them to different pathways of neuronal processing, potentially leading to the plastic reorganization of the network itself. However, this work is the first attempt to use neuromodulatory phase‐encoded MS, mimicking a structured input into the hippocampus circuitry, to relay information from a single assembly being activated to the network‐level organization. There are numerous studies demonstrating the ability of external stimuli to entrain hippocampal theta activity, phase‐locking it to the stimuli. Nevertheless, the neuromodulatory stimuli were either a high‐frequency burst repeating itself within the theta period or external sensory or transcranial alternating current stimulation (tACS) oscillating within the theta range. Still, never a stimulus pattern phase‐coded within a theta period. The fact that the MSf pattern promoted hippocampal plasticity suggests that it was also able to entrain theta, and that MSr patterns failed to do so. Ongoing experiments in our laboratory are attempting to address this issue using simultaneous video‐EEG recordings during memory acquisition and retrieval.

## 5 Conclusion

By evaluating the effect of the stimulation pattern, including the phase of stimulation pulses relative to the overall theta frequency of stimulation, our data indicate a potential role for temporal patterning in engram formation within the hippocampus.

## Author Contributions

**Márcio Flávio Dutra Moraes:** writing – original draft, validation, supervision, resources, project administration, funding acquisition, conceptualization. **Grace Schenatto Pereira:** writing – original draft, validation, supervision, resources, project administration, funding acquisition, conceptualization. **Leonardo de Oliveira Guarnieri:** review and editing, methodology, investigation, data curation. **Paula Gonçalves Vieira Teixeira:** conceptualization, methodology, investigation, data curation.

## Funding

This work was supported by Fundação de Amparo à Pesquisa do Estado de Minas Gerais (APQ‐03295‐18), Conselho Nacional de Desenvolvimento Científico e Tecnológico (309428/2021‐1, 408170/2023‐9), Coordenação de Aperfeiçoamento de Pessoal de Nível Superior, and UFMG partnership with Harvard Medical School.

## Conflicts of Interest

The authors declare no conflicts of interest.

## Data Availability

Data available on our servers and GitHub.
